# Study on the associations of physical activity types and cardiovascular diseases among Chinese population using latent class analysis method

**DOI:** 10.1038/s41598-022-12182-9

**Published:** 2022-05-16

**Authors:** Chong Chen, Jiali Liu, Shurong Lu, Ganling Ding, Jiaqi Wang, Yu Qin, Zengwu Wang, Xin Wang, Zhiyong Zhang, Quanyong Xiang

**Affiliations:** 1 School of Public Health, Southeast University, 87 Dingjiaqiao Road, Nanjing, 210009 Jiangsu Province China; 2 Department of Chronic Non-Communicable Disease Control, Jiangsu Provincial Center for Disease Control and Prevention, 172 Jiangsu Road, Nanjing, 210009 Jiangsu Province China; 3 Division of Prevention and Community Health, National Center for Cardiovascular Disease, 167 Beilishi Road, Beijing, 100037 China; 4 Department of Cardiology, Suqian First Hospital, Suqian Branch of Jiangsu Province Hospital, 120 Suzhi Road, Suqian, 223899 Jiangsu Province China

**Keywords:** Cardiology, Diseases, Risk factors

## Abstract

Previous studies reported on the association between physical activity (PA) and cardiovascular diseases (CVD_S_) among the Western population. However, evidence on the association between different patterns of PA and the risk of CVD_S_ among Chinese population are limited. This study aims to evaluate the association of different PA types and the risk of CVD_S_ in a Chinese adult population. A total of 3568 community residents were recruited from Jiangsu Province of China using a stratified multistage cluster sampling method. The latent class analysis method was employed to identify the types of PA, and the Framingham risk score (FRS) was used to estimate the risk of CVD_S_ within 10 years. Three types of PA were identified: CLASS1 represented participants with high occupational PA and low sedentary PA (32.1% of male, 26.5% of female), ClASS2 represented those engaging in low occupational PA and high leisure-time PA (27.0% of male, 14.2% of female), and CLASS3 represented low leisure-time and high sedentary PA (40.9% of male, 59.3% of female). The average of FRS in males was higher than that in females across PA types. CLASS1 (OR = 0.694, 95%CI 0.553–0.869) and CLASS2 (OR = 0.748, 95%CI 0.573–0.976) were both found to be protective against CVD_S_ in males; however, such associations were not statistically significant among females. Therefore, higher occupational or leisure-time PA appear to be associated with decreased risk of CVD_S_, while more sedentary behaviors may increase the risk of CVD_S_, particularly for male Chinese adults.

## Introduction

Increasing in the number of the aging population and the acceleration of urbanization have significantly increased the prevalence of cardiovascular diseases (CVD_S_), including coronary heart disease, cerebrovascular disease, rheumatic heart disease, and other conditions^1^. According to the *National Report on Cardiovascular Diseases (2018)* in China^2^, the number of patients with CVD_S_in China have reached 290 million. The main causes of CVD_S_ are unhealthy lifestyle behavior and reduced physical activity^3^. Previous studies revealed that regular physical activity (PA) was critical in preventing chronic diseases, including CVD_S_^4,5^. Bennett et al. proposed that PA can be categorized as occupational, commuting, household, and recreational^6^. *The Global Burden of Diseases Report* estimated that low levels of PA accounted for 1.26 million premature deaths and 2.37 million disability-adjusted life-years worldwide in 2017^7^. Meanwhile, high levels of either occupational or leisure-time PA have been found to be associated with a lower risk of CVD_S_ in high-income countries^8^. However, the association between different types of PA and the risk of CVD_S_ among different subgroups of the population in China, so far, have been rarely reported^6,9^.

Latent class analysis (LCA) uses the latent class model (LCM) to explain the relationship between explicit class variables with intrinsic latent class variables^10^. LCA can identify subgroups of people who share common characteristics so that people within the subgroups have a similar scoring pattern on the measured variable, while the difference in scoring patterns between subgroups is as distinctly different as possible^11^. LCA analysis uses a mixture of distributions to identify the most likely model describing the heterogeneity of data as a finite number of classes (subgroups), also known as finite mixture models^12^. LCA was used for modelling the “lifestyle” variable in Miranda’s study to assess the lifestyle of female adolescents based on measurements of behavioral variables^13^. Moreover, in two community samples in Breslau, LCA aimed to empirically examine the structure underlying post-traumatic stress disorder (PTSD) criteria symptoms and identify discrete classes with similar symptom profiles^14^. Similar attempts have also been made in a cohort study, which used data during 2003–2008 from the National Violent Death Reporting System, and included 28,703 suicide decedents from 12 US states^15^. In the present study, we used LCA to estimate the latent PA types of adult residents in Jiangsu province of China and explored the associations of different latent PA types with CVD_S_ risk.

## Materials and methods

### Participants

A multistage stratified cluster sampling method was employed to select participants. Within the seven counties (in rural areas) or districts (in urban areas) of the *Chinese National Disease Surveillance System for Chronic Diseases and Risk Factors* in northern and middle areas of Jiangsu Province of China^16^, five towns /streets were randomly selected from each county/district. Then, two villages/communities were randomly selected from each town/street, followed by sixty households being randomly selected from each village/community. Finally, using the KISH table method, one adult resident aged 18 years or above was selected from each household^17^.

4200 individuals were recruited for participation. We excluded 574 participants whose age did not meet the Framingham Scoring criteria (i.e., 30–74 years old)^18^, 52 participants who had pre-existing CVD_S_, cancer or other severe comorbidities, and 6 participants who did not have complete laboratory data. Finally, a number of 3568 participants were included in this study.

### Questionnaire survey

A standard questionnaire which designed based on the *Questionnaire for the Chinese Chronic Non-communicable Disease and Risk Factor Surveillance* (2010)^16^ was used to collect information on demographic information (i.e., residence, gender, age, educational level, marital status), behavioral factors (i.e., tobacco smoking, alcohol drinking, physical activity and daily sedentary behaviors), and health condition (i.e., hypertension, diabetes, and dyslipidemia). All surveys were conducted face-to-face by interviewers, who had received proper training and passed relevant assessment. The *Global Physical Activity Questionnaire (GPAQ)*^19^ was used to assess the frequency and duration of several components of PA in different components, including: (1) occupational, agriculture, and housework activity; (2) commuting related physical activity; (3) leisure-time physical activity; (4) sedentary behaviors. Levels of agreement with objective measurements indicated that the GPAQ was a valid measure of moderate-to-vigorous physical activities^20^.

### Anthropometric measurements

Height, body weight, waist circumference, and blood pressure were measured by anthropometric investigators using unified brands and models instruments. All investigators successfully completed a training program that introduced them with the specific tools and methods used in this study, as well as with the aims of this study. Briefly speaking, height was measured by a height meter with a maximum range of 2.0 m and a minimum scale of 0.1 cm. The body weight was measured by an electronic scale with a maximum range of 150 kg and an accuracy of 0.1 kg. The waist circumference was measured by a leather tape, which was measured at the midpoint between the lowest rib margin and the lower 12th costal margin. Blood pressure was measured 3 times using an automated device (OMRON HEM-7207)^21^ at the left-arm according to the standard measuring protocol. All sphygmomanometers were calibrated by the manufacturer and checked by the national quality assurance team department. The mean value of the three measurements was used as the final blood pressure values. Details of the anthropometric measurements had been documented elsewhere^22^.

### Blood sample collection and laboratory tests

A volume of 4—5 ml venous blood sample was collected in a vacuum tube containing sodium fluoride in the morning, after overnight fasting of at least 10 h. Fasting plasma glucose (FPG) was measured by glucose oxidase or hexokinase methods within 12 h after collecting in an accredited laboratory. Serum total cholesterol (TC), low-density lipoprotein cholesterol (LDL-C), high-density lipoprotein cholesterol (HDL-C), and triglycerides (TG) were measured using auto-analyzers (Abbott Laboratories) in Jiangsu Province Center for Disease Control and Prevention, which was certificated by The National Laboratory Certification of China.

### Measurement of the risk of CVD_S_

In this study, we used the Framingham Risk Score (FRS) to estimate a person’s chance of developing a CVD_S_ event in the next ten years. The FRS, expressed as a percentage, was calculated based on the prediction equation known as the “Framingham Risk Equation” , which consisted of age, TC, HDL-C, SBP, treatment for hypertension, smoking status, and diabetic status^18^. The risk of CVD_S_ was categorized as: “low” if the FRS ≤ 10%; “intermediate” if the FRS was between 11 and 20%; “high” if the FRS > 20%^23^.

### Classification of physical activity

In this study, the PA of participants was classified using the LCA, an analysis method established on the basis of probability distribution and a log-linear model. It can make up for the traditional statistical methods that only focus on a single variable and play a role of considering the comprehensive effect of multiple factors. The model of LCA was judged using the following test standards^24^: (1) Akaike information criterion (AIC), Bayesian information criterion (BIC), and adjusted Bayesian information criterion (aBIC). The smaller the three indexes, the better the model fitting effect could be; (2) Entropy, the larger the value, the higher the accuracy of the classification could be; (3) In combination with the adjusted Lo-Mendell-Rubin likelihood ratio test (LMR) and the bootstrap-based likelihood ratio test (BLRT), the model of K categories was significantly better than the model of K-1 categories, while it indicates *P* < 0.05 of these indicators. The best classification was determined by considering all above indicators and relevant professional knowledge was used for the interpretation of results.

### Definitions of other involved variables

Body mass index (kg/m^2^) was calculated as weight divided by height squared. Participants were categorized as: underweight (BMI < 18.5 kg/m^2^), normal (18.50 ≤ BMI < 24.00 kg/m^2^), overweight (24.00 ≤ BMI < 28.00 kg/m^2^), and obese (BMI ≥ 28.00 kg/m^2^) according to the standard made by the working group on obesity in China for Chinese population^25^. Central obesity was defined as: males with a waist circumference ≥ 90 cm or females with a waist circumference ≥ 85cm^26^.

Hypertension was defined as having a self-report history of hypertension, receiving BP-lowering treatment, or having an average measured systolic BP of at least 140 mmHg or a diastolic BP of at least 90 mmHg (or both) during the study period^27^.

Diabetes mellitus was defined as FPG ≥ 7.0 mmol/L, or 2-h OGTT ≥ 11.11 mmol/L, or having a self-report history of diabetes, or taking hypoglycemic drugs during the study period^28^. Dyslipidemia was defined as TC ≥ 6.22 mmol/L, and/or TG ≥ 2.26 mmol/L, and/or LDL-C ≥ 4.14 mmol/L, and/or HDL-C ≤ 1.04 mmol/L^29^.

Current smoking was defined as having smoked at least 100 cigarettes, or equivalent other tobacco products in one’s lifetime, and currently smoking cigarettes. Drinking alcohol more than once per month over the past 12 months prior to the interview was defined as current drinking^16^.

### Statistical analysis

General descriptive analysis and χ^2^ test were used to compare the potential differences of categorical variable among groups. The effects of different PA types on the risk of CVD_S_ were analyzed by ordinal logistic regression. Given that age, blood pressure, smoking status, and other factors have been included in the calculation of the FRS, these variables were not adjusted in the ordinal logistic regression analysis. A two-side *P*-value < 0.05 was considered statistically significant. All these analyses were performed using SPSS statistical software (v23.0), while the MPLUS statistical software (v8.0) was used to analyze the potential categories of PA (Latent Classes).

### Ethics approval and consent to participate

Informed written consent was obtained from all participants. The procedures were in accordance with the standards of the ethics committee of Jiangsu Provincial Center for Disease Control and Prevention and with the Declaration of Helsinki (1975, revised 2013). This study protocol was approved by the ethical review committee at the Jiangsu Province Center for Disease Control and Prevention (the committee’s reference number: **SL2017-B002-01**). Individual person’s data have not been contained in any form (including any individual details, images, or videos) in this manuscript.

## Results

### Characteristics of participants

Of the 3568 participants (men, 43.0%), the average age was 52.04 years (SD = 11.08). Compared with females, males had a higher percentage of higher education or having a job. Males were more likely to be smokers, to consume alcohol, or to have hypertension, whilst females were more likely to have central obesity or dyslipidemia (Table 1).

**Table 1:** Characteristics of participants by gender.

	Categories	Total	Female n (%)	Male n (%)	χ^2^	*P*
Age (years)	30–34	196	113 (5.6)	83 (5.4)	25.770	< 0.001
	35–44	835	521 (25.6)	314 (20.5)		
	45–54	954	566 (27.8)	388 (25.3)		
	55–64	1038	559 (27.5)	479 (31.2)		
	65–74	545	275 (13.5)	270 (17.6)		
Education	Primary or below	1999	1333 (65.5)	666 (43.4)	173.690	< 0.001
	Middle	1414	631 (31.0)	783 (51.0)		
	High school or above	155	70 (3.4)	85 (5.5)		
BMI (kg/m^2^)	≤ 18.49	57	30 (1.5)	27 (1.8)	6.385	0.094
	18.50 ~ 23.99	1582	874 (43.1)	708 (46.3)		
	24.00 ~ 27.99	1366	788 (38.8)	578 (37.8)		
	≥ 28.00	552	337 (16.6)	215 (14.1)		
Work	No	824	588 (28.9)	236 (15.4)	90.053	< 0.001
	Yes	2744	1446 (71.1)	1298 (84.6)		
Sleep duration	< 6 h	231	149 (7.3)	82 (5.3)	23.130	< 0.001
	6 ~ 8 h	2514	1369 (67.3)	1145 (74.7)		
	> 8 h	822	516 (25.4)	306 (20.0)		
Smoking	No	2596	1965 (96.6)	631 (41.1)	1359.899	< 0.001
	Not everyday	140	18 (0.9)	122 (8.0)		
	Everyday	832	51 (2.5)	781 (50.9)		
Alcohol drinking	No	2282	1742 (85.6)	540 (35.2)	1052.172	< 0.001
	Before 30 days	274	130 (6.4)	144 (9.4)		
	Within 30 days	1012	162 (8.0)	850 (55.4)		
Overweight or obesity	No	1650	909 (44.7)	741 (48.3)	4.597	0.032
	Yes	1918	1125 (55.3)	793 (51.7)		
Central obesity	No	1651	881 (43.3)	770 (50.2)	16.659	< 0.001
	Yes	1917	1153 (56.7)	764 (49.8)		
Hypertension	No	1693	1044 (51.3)	649 (42.3)	28.532	< 0.001
	Yes	1875	990 (48.7)	885 (57.7)		
Diabetes mellitus	No	3240	1851 (91.0)	1389 (90.5)	0.217	0.641
	Yes	328	183 (9.0)	145 (9.5)		
Dyslipidemia	No	2276	1381 (67.9)	895 (58.3)	34.540	< 0.001
	Yes	1292	653 (32.1)	639 (41.7)		

### Identification of PA types using LCA method

**Figure 1:**
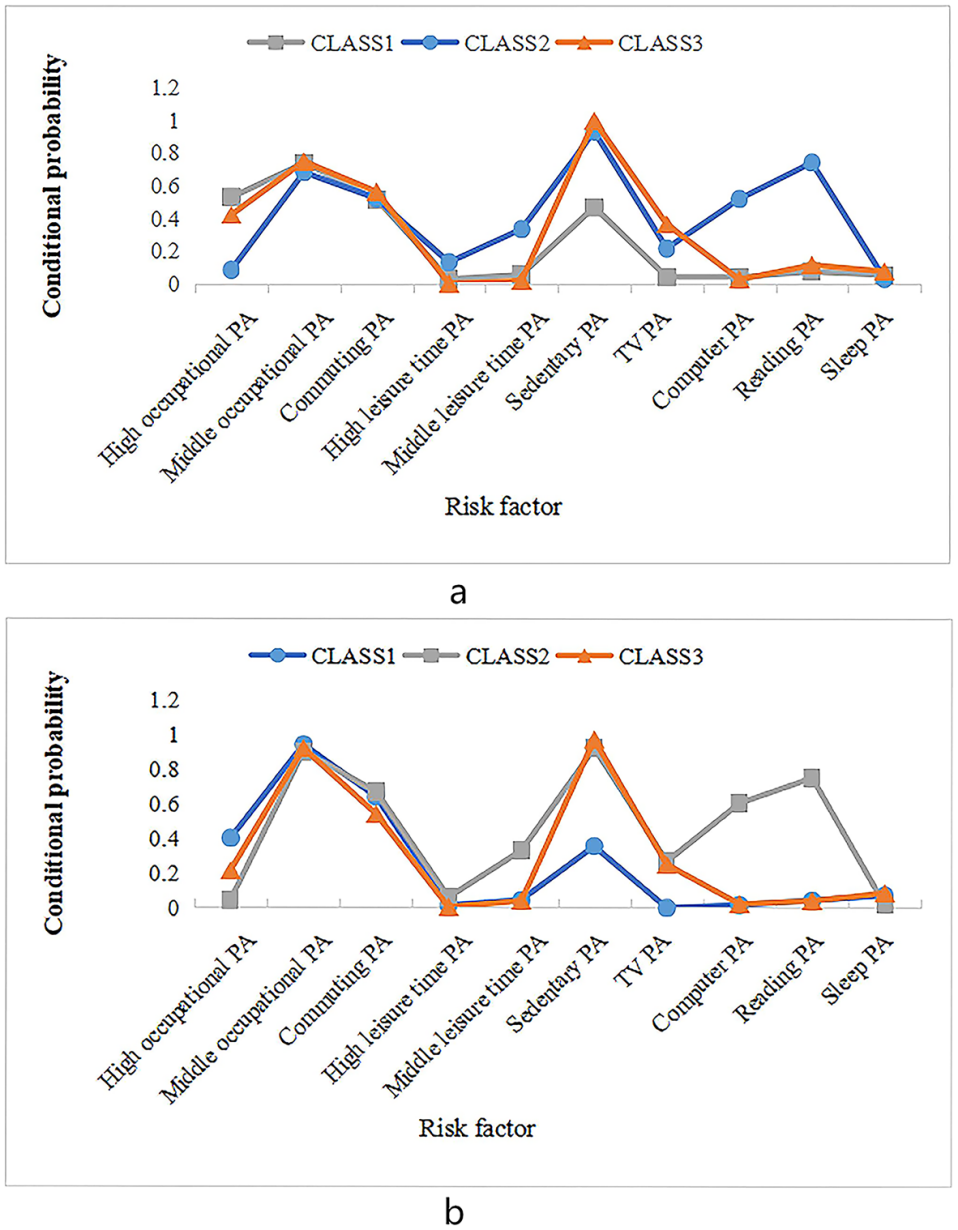
(**a**) Conditional probability distribution for three-category of physical activity for males; (**b**) Conditional probability distribution for three-category of physical activity for females.

In the LCA of PA, 10 variables were included in the GPAQ, including high occupational PA, medium–low occupational PA, commuting PA, high leisure-time PA, medium–low leisure time PA, sedentary PA, TV PA, computer PA, reading PA, and sleeping PA. Five latent class models were fitted for both men and women (Table 2). As was shown in Table 2, with the increase in model categories, Log-like hood (Log (L)), AIC, BIC, and aBIC decreased. In males, BIC value of 3 category model reached the minimum and *P*-value for the LMR was 0.004, however fitting four category model, *P*-value for the LMR was 0.680. Therefore the three category model had the best fitting degree. Similarly,in females the three category model had the best fitting degree. According to the results of the conditional probability distribution of each item in three categories of each gender (Fig. 1), the performance of Latent CLASS1 was high occupational PA, low sedentary PA; the performance of Latent CLASS2 was low occupational and high leisure-time PA; the performance of Latent CLASS3 was low leisure-time PA, high sedentary PA. There were 492 (32.1%), 414 (27.0%) and 628 (40.9%) male participants in these three classifications, respectively, while there were 539 (26.5%), 288 (14.2%) and 1207 (59.3%) female participants, respectively.

**Table 2:** The fitting index of latent category model for different categories.

Model	df		Log(L)	AIC	BIC	aBIC	Entropy	LMR	BLRT	Class probability
Male										
1	11		−7984.588	15,991.176	16,049.868	16,014.924				
2	23		−7684.239	15,414.478	15,537.197	15,464.132	0.696	0.000	0.000	0.265/0.735
3	35		−7636.682	15,343.363	15,530.111	15,418.924	0.547	0.004	0.000	0.270/0.409/0.321
4	47		−7602.892	15,299.784	15,550.559	15,401.252	0.599	0.680	0.000	0.321/0.129/0.375/0.175
5	59		−7574.504	15,267.008	15,581.811	15,394.383	0.650	0.011	0.000	0.062/0.130/0.397/0.118/0.293
Female										
1	11		−8964.428	17,950.856	18,012.652	17,977.704				
2	23		−8637.917	17,321.834	17,451.042	17,377.970	0.797	0.000	0.000	0.149/0.851
3	35		−8543.263	17,156.526	17,353.147	17,241.950	0.614	0.000	0.000	0.265/0.593/0.142
4	47		−8505.732	17,105.463	17,369.498	17,220.176	0.672	0.024	0.000	0.102/0.221/0.542/0.135
5	59		−8481.928	17,081.857	17,413.305	17,225.858	0.560	0.184	0.000	0.481/0.227/0.150/0.028/0.115

### Comparison of the characteristics in different Latent Classes of PA

The baseline characteristics of participants were given by classifications in Tables 3 and 4. There were significant differences in age, education status, marital status, BMI, work status, sleep duration, smoking status, low HDL-C, high TG, hypertension, hyperglycemia and central obesity among the three Latent classes of male PA (*P* < 0.05).There were significant differences in age, education status, marital status, BMI, work status, sleep duration, alcohol consumption, high TG, low HDL-C, Hypertension, and Central obesity among the three Latent classes of female PA (*P* < 0.01).

**Table 3:** Comparison of characteristics among different potential categories of PA for males.

	Categories	N	CLASS1 n (%)	CLASS2 n (%)	CLASS3 n (%)	*χ*^2^	*P*
Age(years)	18 ~ 34	83	22 (26.5)	38 (45.8)	23 (27.7)	111.911	< 0.001
	35 ~ 44	314	84 (26.8)	133 (42.4)	97 (30.9)		
	45 ~ 54	388	142 (36.6)	106 (27.3)	140 (36.1)		
	55 ~ 64	479	183 (38.2)	83 (17.3)	213 (44.5)		
	≥ 65	270	61 (22.6)	54 (20.0)	155 (57.4)		
Education status	Primary school or below	666	264 (39.6)	55 (8.3)	347 (52.1)	337.152	< 0.001
	Middle school	783	226 (28.9)	280 (35.8)	277 (35.4)		
	High school or above	85	2 (2.4)	79 (92.9)	4 (4.7)		
Marital status	Married	1383	450 (32.5)	389 (28.1)	544 (39.3)	16.530	< 0.001
	Unmarried	151	42 (27.8)	25 (16.6)	84 (55.6)		
BMI(kg/m^2^)	≤ 18.49	27	8 (29.6)	9 (33.3)	10 (37.0)	48.892	< 0.001
	18.50 ~ 23.99	708	240 (33.9)	136 (19.2)	332 (46.9)		
	24.00 ~ 27.99	578	181 (31.3)	182 (31.5)	215 (37.2)		
	≥ 28.00	215	62 (28.8)	86 (40.0)	67 (31.2)		
Work	No	236	49 (20.8)	90 (38.1)	97 (41.1)	23.968	< 0.001
	Yes	1298	443 (34.1)	324 (25.0)	531 (40.9)		
Sleep duration	< 6 h	82	19 (23.2)	9 (11.0)	54 (65.9)	50.030	< 0.001
	6 ~ 8 h	1146	336 (29.3)	333 (29.1)	477 (41.6)		
	> 8 h	306	137 (44.8)	72 (23.5)	97 (31.7)		
Smoking	No	631	200 (31.7)	182 (28.8)	249 (39.5)	24.172	< 0.001
	Not everyday	122	36 (29.5)	52 (42.6)	34 (27.9)		
	Everyday	781	256 (32.8)	180 (23.0)	345 (44.2)		
Alcohol drinking	No	540	169 (31.3)	126 (23.3)	245 (45.4)	8.497	0.075
	Before 30 days	144	49 (34.0)	40 (27.8)	55 (38.2)		
	Within 30 days	850	274 (32.2)	248 (29.2)	328 (38.6)		
High TC	Yes	63	20 (4.1)	24 (5.8)	19 (3.0)	4.87	0.088
	No	1471	472 (95.9)	390 (94.2)	609 (97.0)		
High TG	Yes	262	73 (14.8)	92 (22.2)	97 (15.4)	10.661	0.005
	No	1272	419 (85.2)	322 (77.8)	531 (84.6)		
Low HDL-C	Yes	430	115 (23.4)	149 (36.0)	166 (26.4)	19.085	< 0.001
	No	1104	377 (76.6)	265 (64.0)	462 (73.6)		
High LDL-C	Yes	24	6 (1.2)	8 (1.9)	10 (1.6)	0.747	0.688
	No	1510	486 (98.8)	406 (98.1)	618 (98.4)		
Hypertension	Yes	885	265 (53.9)	230 (55.6)	390 (62.1)	8.735	0.013
	No	649	227 (46.1)	184 (44.4)	238 (37.9)		
Hyperglycemia	Yes	145	46 (9.3)	51 (12.3)	48 (7.6)	6.382	0.041
	No	1389	446 (90.7)	363 (87.7)	580 (92.4)		
Central obesity	Yes	764	213 (43.3)	258 (62.3)	293 (46.7)	36.77	< 0.001
	No	770	279 (56.7)	156 (37.7)	335 (53.3)

**Table 4:** Comparison of the distribution of characteristics among different potential categories of PA for females.

	Group	N	CLASS1 n (%)	CLASS2 n (%)	CLASS3 n (%)	*χ*^2^	*P*
Age(years)	18 ~ 34	113	14 (12.4)	51 (45.1)	48 (42.5)	190.908	< 0.001
	35 ~ 44	521	108 (20.7)	120 (23.0)	293 (56.2)		
	45 ~ 54	566	140 (24.7)	70 (12.4)	356 (62.9)		
	55 ~ 64	559	191 (34.2)	38 (6.8)	330 (59.0)		
	≥ 65	275	86 (31.3)	9 (3.3)	180 (65.5)		
Education status	Primary school or below	1333	417 (31.3)	29 (2.2)	887 (66.5)	587.770	< 0.001
	Middle school	631	115 (18.2)	202 (32.0)	314 (49.8)		
	High school or above	70	7 (10.0)	57 (81.4)	6 (8.6)		
Marital status	Married	1805	460 (25.5)	269 (14.9)	1076 (59.6)	12.692	0.002
	Unmarried	229	79 (34.5)	19 (8.3)	131 (57.2)		
BMI (kg·cm^-2^)	≤ 18.49	30	4 (13.3)	3 (10.0)	23 (76.7)	21.571	0.001
	18.50 ~ 23.99	874	237 (27.1)	154 (17.6)	483 (55.3)		
	24.00 ~ 27.99	788	207 (26.3)	94 (11.9)	487 (61.8)		
	≥ 28.00	337	90 (26.7)	35 (10.4)	212 (62.9)		
Work	No	588	156 (26.5)	112 (19.0)	320 (54.4)	19.332	< 0.001
	Yes	1446	383 (26.5)	176 (12.2)	887 (61.3)		
Sleep duration	< 6 h	149	37 (24.8)	4 (2.7)	108 (72.5)	86.996	< 0.001
	6 ~ 8 h	1369	301 (22.0)	241 (17.6)	827 (60.4)		
	> 8 h	516	201 (39.0)	43 (8.3)	272 (52.7)		
Smoking	No	1965	526 (26.8)	283 (14.4)	1156 (58.8)	6.701	0.153
	Not everyday	18	3 (16.7)	1 (5.6)	14 (77.8)		
	Everyday	51	10 (19.6)	4 (7.8)	37 (72.5)		
Alcohol drinking	No	1742	458 (26.3)	204 (11.7)	1080 (62.0)	66.394	< 0.001
	Before 30 days	130	38 (29.2)	38 (29.2)	54 (41.5)		
	Within 30 days	162	43 (26.5)	46 (28.4)	73 (45.1)		
High TC	Yes	70	17 (3.2)	9 (3.1)	44 (3.6)	0.372	0.83
	No	1964	522 (96.8)	279 (96.9)	1163 (96.4)		
High TG	Yes	266	71 (13.2)	18 (6.3)	177 (14.7)	14.488	0.001
	No	1768	468 (86.8)	270 (93.8)	1030 (85.3)		
Low HDL-C	Yes	362	68 (12.6)	70 (24.3)	224 (18.6)	18.707	< 0.001
	No	1672	471 (87.4)	218 (75.7)	983 (81.4)		
High LDL-C	Yes	27	9 (1.7)	3 (1.0)	15 (1.2)	0.728	0.695
	No	1007	530 (98.3)	285 (99.0)	1192 (98.8)		
Hypertension	Yes	990	302 (56.0)	76 (26.4)	612 (50.7)	70.917	< 0.001
	No	1044	237 (44.0)	212 (73.6)	595 (49.3)		
Hyperglycemia	Yes	183	50 (9.3)	23 (8.0)	110 (9.1)	0.431	0.806
	No	1851	489 (90.7)	265 (92.0)	1097 (90.9)		
Central obesity	Yes	1153	306 (56.8)	122 (10.6)	725 (60.1)	29.688	< 0.001
	No	881	233 (43.2)	166 (57.6)	482 (39.9)		

### Relationships between PA types and the risk of CVD_S_

Comparison analysis among the three PA types in males revealed significant differences in their 10-year FRS. As shown in Table 5, the FRSs of males were higher than that of females. Among males, the FRS for CLASS1 and CLASS2 were lower than that of CLASS3, which had the largest number of participants. CLASS1 (*OR* = 0.654,95%CI 0.526–0.813) and CLASS2 (*OR* = 0.544, 95%CI 0.432–0.685) were found to be protective against the risk of CVD_S_ compared to CLASS3. After adjusting for potential confounding factors, the relationship between CLASS 1(OR = 0.694, 95%CI 0.553–0.869) and CLASS 2(OR = 0.748, 95%CI 0.573–0.976) and the risk CVD_S_ was slightly attenuated but remained statistically significant. Among females, CLASS2 was inversely correlated with CVD_S_ (*OR* = 0.451, 95%CI 0.316–0.643), but such association disappeared after adjusted for potential confounders.

**Table 5:** FRS among different PA types and associations of PA types and the risk of CVD_S_.

Latent class	N	FRS(%)^#^	Crude		Adjusted*	
			*P*	*OR* (95%*CI*)	*P*	*OR *(95%*CI*)
Male	1534					
CLASS1	492	13.48(7.23,23.17)	< 0.001	0.654 (0.526–0.813)	0.002	0.694 (0.553–0.869)
CLASS2	414	11.22(5.58,23.57)	< 0.001	0.544 (0.432–0.685)	0.032	0.748 (0.573–0.976)
CLASS3	628	17.10(9.17,28.41)	Ref			
Female	2034					
CLASS1	539	5.42(2.58,11.06)	0.683	1.048 (0.836–1.314)	0.732	1.042 (0.823–1.319)
CLASS2	288	2.67(1.32,6.04)	< 0.001	0.451 (0.316–0.643)	0.607	0.896 (0.588–1.363)
CLASS3	1207	5.23(2.53,10.55)	Ref			

*Adjusted for education, work, drinking, BMI, dyslipidemia, central obesity, and overweight/obesity; ^#^median (25th percentile, 75th percentile).

## Discussion

The China Kadoorie Biobank (CKB) study^30^ reported that total levels of PA was strongly, and inversely, associated with CVD_S_-related mortality in Chinese population^31^. Like in many other developed countries, the standard of living in China greatly improved, leading to drastic lifestyle changes, for example, transferring from a labor-intensive lifestyle to a sedentary lifestyle^3^. A prospective cohort study of 487,334 subjects conducted by Bennett et al^6^ in 10 regions of China showed that higher occupational or non-occupational PA was significantly associated with a lower risk of major CVD_S_ events among Chinese adults. In this study, we classified PA in three groups (Latent Classes), i.e., CLASS1 (high occupational and low sedentary PA), CLASS2 (low occupational and high leisure-time PA), and CLASS3 (low leisure-time and high sedentary PA). Several previous LCA studies provided limited and inconsistent findings in different fields, such as sociology, biology, medicine, and psychology^32^. To the best of our knowledge, this is among the first studies to explore the associations between CVD_S_ and PA types using LCA among Chinese adults with representative data.

This study found that CLASS3 accounted for a big proportion in the three categories of PA (40.9% of males and 59.3% of females). CLASS3 was manifested as high sedentary and low leisure-time activity behavior. A previous survey of nine provinces in China from 1991 to 2011^33^ found that for both adult men and women in China, occupational and domestic PA were the largest contributors to the total PA; meanwhile, this study also revealed that the overall PA of community residents significantly declined in the two decades, and active leisure and travel PA were fairly low. Some studies have shown that the occupational PA, rather than the leisure PA, is the main source for total daily PA^34,35^. Inadequate total daily PA has become one of the major risk factors for China's CVD_S_ death and disease burden^36^. Similarly, physical inactivity and obesity are the biggest public health threats, with 53.5% of adults being physically inactive in Canada^37^. Sedentary PA is also a threat to Americans' physical health, which is why the *2018 Physical Activity Guidelines for Americans, 2nd* edition highlights the shift from sitting time to being more active, ideally by doing moderate- or vigorous-intensity physical activity^38^.

This study explored the relationship of 10-year risk of CVD_S_ predicted by the Framingham risk scoring system with three types of PA . The current data demonstrated that the 10-year risk of CVD_S_ incidence was higher in males compared to females in across the three categories. Previous studies indicated that males had a higher risk to have CVD_S_ events, which may be related to differences in exposure levels, sensitivities of risk factors for CVD_S_ between genders, and sex hormone differences^39,40^. In this study, CLASS3 was associated with higher CVD_S_ risk in both genders compared to CLASS1 and CLASS2. The CLASS1(OR = 0.694, 95%CI 0.553–0.869)and CLASS2(OR = 0.748, 95%CI 0.573–0.976) were found to be related to lower risk of CVD_S_ with 10-year. These results were consistent with previous studies^8,41^. As a result, the 2018 PA guidelines for Americans^38^ emphasize that increasing PA and reducing sedentary time are appropriate for all populations and that even a little increase in PA can bring health benefits. In addition, the American college of sports medicine (ACSM)^42^ suggest that regular PA (for example, exercise, cycling) may reduce insulin levels and renal sympathetic nerve tension by sodium retention and foundation, vasodilator substances by skeletal muscle release cycle, and improve blood pressure, blood lipid, blood glucose and other risk factors of CVD_S_^43^.

The LCA method takes into account the comprehensive effect of multiple factors. It can reveal the characteristics of various groups of people and provide a scientific basis for the designation of targeted intervention and prevention measures. However, several limitations of the study should be considered. First of all, the LCA takes the qualitative data into consideration instead of the comprehensive analysis of its frequency and duration. Second, in this study, a questionnaire survey was used to collect physical activity information, rather than using objective measurements (e.g., pedometers to calculate the exact daily steps), which may lead to recall bias. Nevertheless, the use of a tool with proven validity and reliability, i.e., the GPAQ, together with adequate staff training, can minimize such bias. Third, the FRS was used to estimate the 10-year CVD_S_ risk in this study, which may has neglected important information on the possible effects of ethnicity on the findings. As this was a cross-sectional study, the causal relationship between PA and the risk of CVD_S_ could be hardly established. Consequently, further longitudinal research with robust design is warranted to test this relationship.

To summarize, results from this study revealed potential associations between CVD_S_ and PA types among Chinese adults. Lower occupational and leisure-time PA and higher sedentary PA were associated with increased risk of CVD_S_. Accordingly, we suggest relevant sectors in China to strengthen evidence-based interventions in order to increase the levels of PA of people and reduce the time of sedentary behaviors. Findings from this study can be used to advance public health, particularly in the management of public policies that promote PA and bring more health benefits.

## Data Availability

The datasets used and/or analyzed during the current study are available from the corresponding author upon request.

## Abbreviations

CVD_S_: Cardiovascular diseases
PA: Physical activity
LCA: Latent class analysis
FRS: Framingham risk score
LCM: Latent class model
PTSD: Posttraumatic stress disorder
GPAQ: Global physical activity questionnaire
FPG: Fasting plasma glucose
TC: Serum Total cholesterol
LDL-C: Low-density lipoprotein cholesterol
HDL-C: High-density lipoprotein cholesterol
TG: Triglycerides
AIC: Akaike information criterion
BIC: Bayesian information criterion
aBIC: Adjusted Bayesian information criterion
LMR: Lo-Mendell-Rubin likelihood ratio test
BLRT: Bootstrap-based likelihood ratio test
SBP: Systolic blood pressure
DBP: Diastolic blood pressure
Log (L): Log-like hood
CKB: China Kadoorie Biobank
